# Teaching in radical prostatectomy is safe: a multicenter prospective registry analysis

**DOI:** 10.1007/s11701-026-03350-4

**Published:** 2026-03-30

**Authors:** Nicola Giudici, Raphael Röthlisberger, Nicolas Arnold, Daniel Phat Nguyen, Agostino Mattei, Massimo Valerio, Daniel Eberli, Dominik Abt, Räto Strebel, Daniel Engeler, George Thalmann, Swiss Urology Registry Collaborative Group, Laila Schneidewind, Beat Roth

**Affiliations:** 1 Department of Urology, University Hospital of Bern, Bern, Switzerland; 2 Department of Urology, Réseau Hospitalier Neuchâtelois, Neuchâtel, Switzerland; 3 Department of Urology, University of Lucerne, Luzerner Kantonsspital, Lucerne, Switzerland; 4 Division of Urology, Geneva University Hospitals, Geneva, Switzerland; 5 Department of Urology, University Hospital Zurich, Zurich, Switzerland; 6 Department of Urology, Spitalzentrum Biel, Centre Hospitalier Bienne, Biel, Switzerland; 7 Department of Urology, Cantonal Hospital of Graubünden, Chur, Switzerland; 8 Department of Urology, Cantonal Hospital of St. Gallen, St. Gallen, Switzerland

**Keywords:** Complications, Education, Prostatectomy, Teaching, Trainee

## Abstract

To evaluate the impact of teaching during radical prostatectomy in a nationwide prospective registry, assessing whether teaching influences perioperative or pathological outcomes. Data were extracted from the Swiss Urology Prostatectomy Registry, a nationwide quality-monitoring program including all certified training centers. Teaching was defined as procedures performed by a supervised trainee as primary operator. Baseline and perioperative outcomes were compared descriptively, and multivariable regression models assessed associations between teaching status and perioperative and pathological outcomes. A total of 7,607 men underwent radical prostatectomy between 2020 and 2025, of whom 825 (10.8%) were teaching cases. In multivariable analyses, teaching operations were associated with a higher likelihood of any 30-day complication (OR 1.41; 95% CI 1.09–1.82; *p* = 0.008) and longer operative time, with an adjusted mean increase of 31 min (95% CI 26–37; *p* < 0.001). Teaching status was not associated with severe complications (Clavien–Dindo ≥ III; OR 0.62; 95% CI 0.24–1.36; *p* = 0.283), transfusion requirement (OR 1.56; 95% CI 0.36–4.60; *p* = 0.478), positive surgical margins (OR 0.99; 95% CI 0.81–1.22; *p* = 0.960), nerve-sparing (OR 1.12; 95% CI 0.90–1.39; *p* = 0.306), or lymph node yield (adjusted mean difference + 0.5 nodes; 95% CI − 0.3 to 1.4; *p* = 0.226). Teaching during radical prostatectomy is safe and does not compromise surgical or pathological outcomes. Although longer operative times were observed, supervised trainee involvement can be safely integrated into structured training programs.

**Supplementary Information:**

The online version contains supplementary material available at https://doi.org/10.1007/s11701-026-03350-4.

## Introduction

Prostate cancer (PCa) is the most frequently diagnosed malignancy among men in Europe and remains a leading cause of cancer-related death. Globally, it accounts for over 1.4 million new cases and 375,000 deaths annually [1, 2]. Radical prostatectomy (RP) remains a cornerstone of curative treatment for localized prostate cancer, and with robotic-assisted laparoscopic prostatectomy increasingly being used, ensuring quality and safety during teaching operations has become a critical priority in modern urologic training [3–5]. 

Teaching in surgery is essential to sustain expertise across generations. However, trainee involvement raises concerns about longer operative times and potential compromise of perioperative or oncologic outcomes [6–8]. While several studies suggest that supervised teaching operations in RP are safe and do not appear to compromise major perioperative or oncologic outcomes, the existing evidence arises from single-center, retrospective cohorts, often lacking standardized definitions of trainee participation, or uniform complication grading [9–11]. 

Assessing the safety of teaching during RP is therefore crucial. Transparent benchmarking of teaching versus non-teaching procedures supports national training standards, quality, and patient trust. This multicenter, prospective registry-based study from the Swiss Society of Urology (SWISS UROLOGY) aims to provide robust, real-world evidence on whether teaching in RP affects perioperative or pathological outcomes. The nationwide coverage, standardized prospective data collection, and longitudinal follow-up offer a unique opportunity to objectively evaluate surgical education quality at a national level. To our knowledge, this represents the largest cohort analyzed to date using a high-quality, prospective registry specifically addressing the impact of surgical teaching in RP, focusing on short-term perioperative and pathological outcomes, including complications, blood loss, operative time, transfusion, nerve-sparing, lymph node yield, and positive surgical margins, stratified by teaching and non-teaching procedures.

## Materials and methods

### Data source

This study is based on the national, prospective prostatectomy registry of SWISS UROLOGY. The registry was established in 2020 as part of a quality-monitoring initiative that also includes cystectomy and nephrectomy registries, aiming to assess perioperative outcomes across Switzerland. Participation has progressively expanded: between 2020 and 2024, the registry captured approximately 40% to 50% of all prostatectomies performed nationwide, contributed by 29 centers, around half of all surgical providers in the country, covering all university hospitals as well as the major cantonal and private clinics in Switzerland. Since 1 July 2025, participation has become mandatory for all certified urological training centers, increasing national coverage to roughly 90% of all prostatectomies.

Data are entered into a standardized electronic platform (Adjumed, Zurich, Switzerland) using a prospective, two-step data capture process. Operative and perioperative variables are recorded at the time of surgery by the responsible surgeon, while follow-up data and postoperative outcomes are updated periodically by a dedicated study nurse as part of routine registry follow-up. All data are pseudonymized before central aggregation. The registry systematically records patient-, tumor-, treatment-, and outcome-related variables. Longitudinal follow-up at 3, 12, and 24 months captures PSA kinetics, complications, adjuvant therapy, and survival outcomes. Data quality is ensured by automatic plausibility checks at entry and periodic audits coordinated by SWISS UROLOGY.

The protocol was approved by the Cantonal Ethics Committee of Zurich (BASEC-Nr. Req-2017-00283).

### Exposure and outcomes

The main exposure of interest was whether the procedure was performed as a teaching operation. Teaching procedures were defined as cases in which a surgeon in training acted as the primary operator for the majority of the procedure under the direct supervision of a board-certified attending surgeon. The supervising surgeon remained present in the operating room and could assist or temporarily take over specific operative steps when necessary to ensure patient safety. This classification is a mandatory variable in the registry and was prospectively recorded by the responsible surgeon for every procedure at the time of data entry, ensuring complete exposure information for all patients. Primary outcomes were: intraoperative or postoperative 30-day complications, combined; severe complications (Clavien–Dindo classification ≥ III); transfusion requirement; Positive surgical margin (PSM); PSM in the cT3–T4 subgroup; nerve-sparing performed; amount of lymph node yield; operative time.

### Missing data

Missing data were minimal for most adjustment covariates (≤ 2% for age, BMI, ASA (American Society of Anesthesiologists) Score, D’Amico risk group, prostate volume, surgical technique, lymphadenectomy status, and surgeon classification). Given the low level of missingness, a complete-case analysis was performed, resulting in the exclusion of 311 patients from the adjusted analyses.

### Statistical analysis

Baseline characteristics and unadjusted outcomes were compared between teaching and non-teaching operations. Continuous variables were summarized as median (interquartile range [IQR]), while categorical variables were summarized as counts and percentages. Group comparisons were performed using the Mann–Whitney U test for continuous variables and the chi-square test or Fisher’s exact test, as appropriate, for categorical variables. Binary outcomes, including any 30-day postoperative complication, severe complications (Clavien–Dindo ≥ III), transfusion requirement, positive surgical margins (overall and in the cT3–T4 subgroup), nerve-sparing, were analyzed using multivariable logistic regression models, with results reported as adjusted odds ratios (ORs) and 95% confidence intervals (CIs). Continuous outcomes were analyzed using multivariable linear regression, with regression coefficients representing adjusted mean differences expressed in minutes for operative duration and in number of nodes for lymph node yield among patients undergoing pelvic lymph node dissection. All multivariable models were adjusted for clinically relevant covariates, including age, body mass index, ASA score, D’Amico risk group, prostate volume, surgical technique (open/converted vs. robotic-assisted), pelvic lymph node dissection, and hospital type (university vs. non-university), as appropriate for each outcome. Complete-case analysis was used for all multivariable models. Two-sided p-values < 0.05 were considered statistically significant.

All analyses were performed in R version 4.4.1 (R Foundation for Statistical Computing, Vienna, Austria).

## Results

### Baseline characteristics

**Fig. 1:**
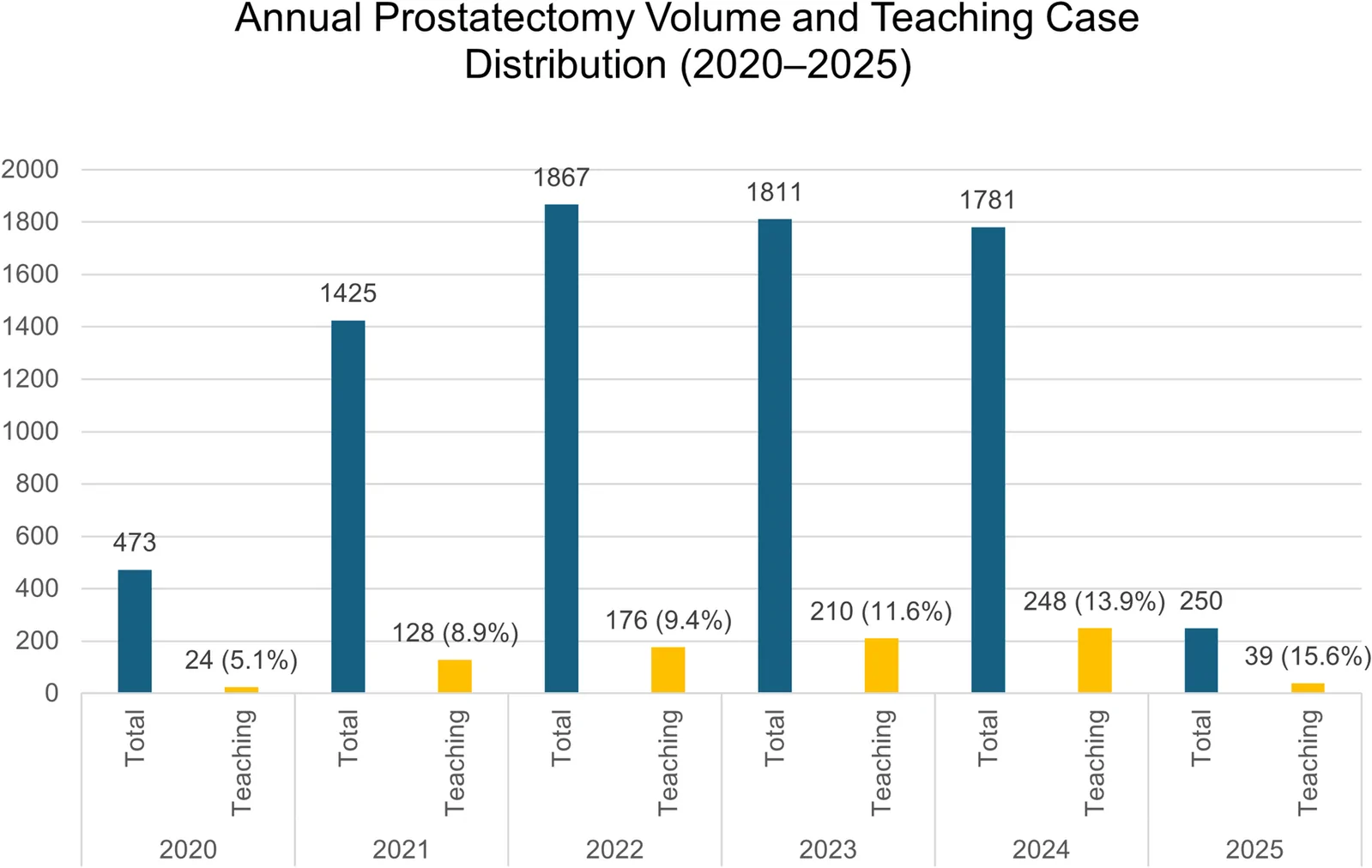
Annual Prostatectomy Volume and Teaching Cases Distribution (2020-2025)

Data from 7,607 men who underwent RP were included in the analysis; 825 (10.8%) procedures were performed as teaching operations. Baseline characteristics are summarized in Table 1. Temporal trends in annual prostatectomy volume and the proportion of teaching cases from 2020 to 2025 are shown in Fig. 1. Over the study period, the proportion of teaching cases steadily increased, rising from 5.1% in 2020 to 15.6% in 2025. Teaching cases were more often performed at university hospitals, and patients in these cases were slightly younger. BMI, ASA class, and comorbidity profiles were broadly comparable between groups. Preoperative PSA and prostate volume showed no meaningful differences. D’Amico risk categories and preoperative ISUP (International Society of Urological Pathology) grade distributions were also similar, with most patients presenting with intermediate- or high-risk disease.

**Table 1:** Patient, tumor, and operative characteristics by teaching status

Variable	Non-teaching (*N* = 6,782)	Teaching (*N* = 825)	*p*-value
**General characteristics**			
**Hospital type**,** n (%)**			< 0.001
University hospital	348 (49.2%)	360 (50.8%)	
Other hospital	6,434 (93.3%)	465 (6.7%)	
Age (years), median [IQR]	67 [62–71]	66 [60–71]	< 0.001
Body mass index (kg/m²), median [IQR]	26.2 [24.2–28.7]	26.5 [24.3–29.0]	0.301
**ASA-score**,** n (%)**			0.013
1	452 (7.4%)	29 (4.2%)	
2	3,981 (65%)	484 (70%)	
3	1,695 (28%)	178 (26%)	
Cardiac comorbidity, n (%)	703 (10%)	72 (8.7%)	0.159
Vascular disease, n (%)	171 (2.5%)	27 (3.3%)	0.244
Pulmonary disease, n (%)	263 (3.9%)	22 (2.7%)	0.103
Renal insufficiency, n (%)	157 (2.3%)	17 (2.1%)	0.735
Diabetes mellitus, n (%)	578 (8.5%)	65 (7.9%)	0.575
Other comorbidities, n (%)	4,054 (60%)	396 (48%)	< 0.001
Length of hospital stay (days), median [IQR]	6 [4–8]	6 [4–8]	0.143
**Preoperative characteristics**			
Preoperative PSA (ng/mL), median [IQR]	7.1 [5.0-10.6]	7.4 [5.0-10.3]	0.800
**Preoperative ISUP grade group**,** n (%)**			0.008
1	1,094 (16%)	95 (12%)	
2	2,925 (43%)	359 (44%)	
3	1,353 (20%)	185 (22%)	
4	1,055 (16%)	138 (17%)	
5	330 (4.9%)	47 (5.7%)	
**D’Amico risk group**,** n (%)**			0.133
Low risk	363 (5.4%)	44 (5.4%)	
Intermediate risk	3,972 (59%)	514 (63%)	
High risk	2,344 (35%)	258 (32%)	
Prostate volume (mL), median [IQR]	41 [31–55]	40 [31–52]	0.022
**Operative characteristics**			
**Surgical technique**,** n (%)**			0.210
Robotic-assisted / Laparoscopic	6,178 (91%)	747 (91%)	
Open / Converted	604 (9%)	78 (9%)	
Pelvic lymph-node dissection, n (%)	5,279 (78%)	553 (67%)	< 0.001
Nerve-sparing performed, n (%)	4,922 (74%)	607 (74%)	1.000
Estimated blood loss (mL), median [IQR]	200 [100–300]	200 [100–350]	0.405
Need for transfusion, n (%)	34 (0.5%)	4 (0.5%)	1.000
Operative time (min), median [IQR]	186 [150–233]	240 [201–277]	< 0.001
**Postoperative and pathologic outcomes**			
Positive surgical margin, n (%)	1,607 (24%)	178 (22%)	0.189
Number of lymph nodes examined, median [IQR]	11 [4–17]	11 [0–18]	0.072
**Postoperative ISUP grade group**,** n (%)**			0.092
1	563 (8.4%)	68 (8.3%)	
2	3,466 (51%)	387 (47%)	
3	1,814 (27%)	241 (29%)	
4	454 (6.7%)	56 (6.8%)	
5	434 (6.4%)	68 (8.3%)	
**Pathologic T stage**,** n (%)**			0.875
pT2	4,371 (64%)	523 (63%)	
pT3	2,354 (35%)	295 (36%)	
pT4	19 (0.3%)	3 (0.4%)	
**Pathologic N stage**,** n (%)**			0.740
pN0	5,863 (86%)	721 (87%)	
pN1	469 (6.9%)	52 (6.3%)	
**Complications**			
Intraoperative complications, n (%)	92 (1.4%)	14 (1.7%)	0.431
Vascular injury	15 (0.2%)	0 (0%)	0.396
Intestinal injury	14 (0.2%)	1 (0.1%)	1.000
Others	62 (0.9%)	13 (1.6%)	0.069
**Postoperative complications**,** n (%)**	746 (11%)	159 (19%)	< 0.001
**Highest Clavien–Dindo grade**,** n (%)**			< 0.001
I	165 (2.4%)	43 (5.2%)	
II	124 (1.8%)	19 (2.3%)	
III	90 (1.3%)	6 (0.7%)	
IV	16 (0.2%)	2 (0.2%)	
V	3 (< 0.1%)	1 (0.1%)	
Urinary infection	168 (2.5%)	56 (6.8%)	< 0.001
Non-urinary infection	90 (1.3%)	22 (2.7%)	0.003
Thromboembolic events	30 (0.4%)	9 (1.1%)	0.032
Neurological	26 (0.4%)	3 (0.4%)	1.000
Cardiac ischemic events	18 (0.3%)	2 (0.2%)	1.000
Cardiac non-ischemic events	26 (0.4%)	5 (0.6%)	0.377
Gastrointestinal	73 (1.1%)	7 (0.8%)	0.545
Pulmonary	23 (0.3%)	8 (1.0%)	0.015
Urogenital/renal	228 (3.4%)	32 (3.9%)	0.440
Wound healing disorders	33 (0.5%)	8 (1.0%)	0.079
Bleeding	97 (1.4%)	21 (2.5%)	0.014
Lymphocele	4 (< 0.1%)	0 (0%)	1.000
Others	182 (2.7%)	37 (4.5%)	0.003

### Perioperative and oncologic characteristics by teaching status

The robotic-assisted approach was used in 91.0% of procedures in both subcohorts, indicating a nearly identical distribution of surgical approach between groups. Teaching operations were associated with a longer median operative time (240 min [IQR 201–277] vs. 186 min [IQR 150–233]). Median estimated blood loss was 200 mL in both groups, with comparable interquartile ranges. A pelvic lymph node dissection (PLND) was performed in 67.0% (95% CI, 63.6–70.2) of teaching cases and 78.0% (95% CI, 77.0–79.0) of non-teaching cases; the amount of lymph node yield was similar between groups (median 11 [IQR 0–18] vs. 11 [IQR 4–17]). Nerve-sparing, whether unilateral or bilateral, was performed in 74% of both teaching and non-teaching operations. Median length of hospital stay was identical across groups (6 days [IQR 4–8]). Rates of positive surgical margins (PSM) were comparable, occurring in 22.0% (95% CI, 19.0–25.3) of teaching cases and 24.0% (95% CI, 23.0–25.0) of non-teaching cases. The postoperative ISUP grade group and pathologic staging (pT, pN) distributions were balanced. Most cases were classified as pT2–3 disease (99%) and node-negative (87%), without relevant differences between teaching and non-teaching operations. Intraoperative complications were rare and occurred at similar rates in teaching and non-teaching operations (1.7% vs. 1.4%). Postoperative complications were more frequent in teaching cases (19% vs. 11%), primarily driven by low-grade events, while severe complications (Clavien–Dindo ≥ III) remained uncommon and comparable between groups. Infectious complications, particularly urinary tract infections, were more frequently observed in teaching operations, whereas transfusion requirements and other complication categories were rare and showed no relevant differences.

### Adjusted perioperative and pathological outcomes

Risk-adjusted odds ratios (ORs), 95% confidence intervals (CIs), and p-values for perioperative and pathologic binary outcomes are summarized in Table 2. Adjusted mean differences for continuous outcomes, including operative time and lymph node yield, derived from multivariable linear regression models are presented in Table 3. Full multivariable regression models for all perioperative and pathological outcomes are provided in Supplementary Tables S2–S9. In the multivariable analysis, teaching operations were independently associated with a higher likelihood of any 30-day complication (OR 1.41; 95% CI, 1.09–1.82; *p* = 0.008). Teaching status, however, was not significantly associated with severe complications (Clavien–Dindo ≥ III; OR 0.62; 95% CI, 0.24–1.36; *p* = 0.283), transfusion requirement (OR 1.56; 95% CI, 0.36–4.60; *p* = 0.478), overall positive surgical margins (OR 0.99; 95% CI, 0.81–1.22; *p* = 0.960), positive margins in the cT3–T4 subgroup (OR 1.23; 95% CI, 0.69–2.18; *p* = 0.478), or the likelihood of nerve-sparing (OR 1.12; 95% CI, 0.90–1.39; *p* = 0.306). Teaching operations were associated with a significantly longer operative time, corresponding to an adjusted mean increase of 31 min (95% CI, 26–37; *p* < 0.001). In contrast, lymph node yield, analyzed among patients undergoing pelvic lymph node dissection, did not differ significantly by teaching status (adjusted mean difference + 0.5 nodes; 95% CI, − 0.3 to 1.4; *p* = 0.226).

**Table 2:** Summary of Adjusted Odds Ratios for Teaching vs. Non-Teaching Operations Across All Outcomes

Outcome	Adjusted OR (Teaching vs. Non-Teaching)	95% CI	*p*-value
Any 30-Day Complication	1.41	1.09–1.82	0.008
Severe Complication (Clavien ≥ III)	0.62	0.24–1.36	0.283
Need for Transfusion	1.56	0.36–4.60	0.478
Positive Surgical Margins	0.99	0.81–1.22	0.960
Nerve-Sparing Performed	1.12	0.90–1.39	0.306
Positive Surgical Margins in cT3–T4	1.23	0.69–2.18	0.478

**Table 3:** Adjusted Mean Differences for Operative Time and Lymph-Node Yield

Outcome	Adjusted Mean Differences (Teaching vs. Non-Teaching)	95% CI	*p*-value
Operative Time (minutes)	+ 31	26–37	< 0.001
Lymph-Node Yield (nodes)	+ 0.5	−0.3–1.4	0.226

## Discussion

In this large, multicenter analysis from the prospective Swiss national prostatectomy registry, teaching operations accounted for approximately 11% of all radical prostatectomies (RP). Teaching procedures were associated with a higher likelihood of overall 30-day complications and longer operative times. However, when focusing solely on severe complications (Clavien–Dindo ≥ III), no significant association between teaching status and severe postoperative morbidity was observed. Notably, the majority of recorded complications were low-grade events, predominantly urinary tract infections, which are unlikely to be clinically consequential. Due to the nature of the registry, perioperative antibiotic management and preoperative infection screening were not captured, and variations in antibiotic prophylaxis strategies across participating centers could potentially have influenced the observed rates of urinary tract infections. Accordingly, the absence of an association with severe complications represents the more clinically relevant finding. Transfusion rates and median blood loss were identical between groups, indicating no clinically meaningful difference in bleeding risk. Furthermore, positive surgical margins, lymph node yield, and nerve-sparing rates were comparable between teaching and non-teaching cases.

Our findings are consistent with previous studies demonstrating that trainee participation in RP modestly increases operative duration but does not compromise surgical safety or pathological outcomes [9]. Thomas et al. analyzed 1,019 robotic-assisted RP and found that trainee involvement led to longer operative times (241 vs. 200 min, *p* < 0.001) and slightly higher blood loss (190 vs. 120 mL, *p* < 0.001) without increasing transfusion, complication, or positive margin rates [12]. Similarly, Chen et al. reported that console time was longer when trainees performed key steps (137 vs. 120 min, *p* = 0.022) but that estimated blood loss, margin status, and complication rates were unaffected [13]. Notably, in our nationwide cohort, teaching operations were associated with an increase of 31 min in operative time, a magnitude comparable to that reported in prior single-center series. In relative terms, this corresponds to roughly a 15% prolongation of total operative duration. While this increase is measurable and operationally relevant, it is unlikely to be clinically meaningful for individual patients, particularly in light of the absence of adverse effects on severe complications and pathological outcomes. From a practical perspective, this additional time should be taken into account in operating room scheduling and institutional planning. More broadly, prolonged operative duration has been associated with increased perioperative morbidity in the surgical literature, supporting the importance of minimizing unnecessary delays whenever possible [14]. Importantly, our prospective, national registry design extends these earlier observations by encompassing both academic and non-academic centers and a substantially larger and more representative patient population.

Although some other surgical contexts have reported less favorable outcomes with trainee participation, including general, vascular, and orthopedic procedures, these findings likely reflect differences in procedural complexity, standardization, and supervision rather than the act of teaching itself [15–18]. In our cohort, open procedures accounted for around 10% of cases and were evenly distributed between teaching and non-teaching operations, further supporting that differences in surgical access are unlikely to explain the observed outcomes. In the setting of RP, especially robotic-assisted, the procedure is highly standardized, modular and stepwise (with initial simpler segments and progressively more challenging ones), making it an ideal supervised learning environment that allows trainees to gain competence without compromising precision or pathological control.

A notable finding of this study is that half of all procedures performed at university hospitals were teaching operations (50.8%), whereas in non-university hospitals this was the case in only 6.7%, reflecting the central role of academic centers in surgical training. This concentration of teaching cases in university hospitals is not surprising; however, it warrants reflection. Teaching outcomes depend not only on supervision and trainee skill but also on whether the institutional setting provides the infrastructure, case volume, and standardized processes that enable safe training. These findings reinforce that structured, supervised teaching during RP can be safely integrated into clinical practice without compromising major surgical or pathological outcomes. Importantly, because university and non-university hospitals may differ in perioperative infrastructure, team experience, and care pathways, hospital type was included as a covariate in the multivariable analyses to account for potential institutional differences that could independently influence outcomes.

The observed increases in minor complications and operative time are expected within the context of surgical training, provided that appropriate case selection and oversight are ensured. Institutions should continue to promote active trainee participation as an integral component of maintaining procedural expertise and ensuring workforce sustainability in urology. Transparent benchmarking of outcomes, such as that enabled by national registries, is valuable for monitoring performance, optimizing training strategies, and reassuring patients regarding the safety of teaching environments. In this context, structured, proficiency-based robotic training programs, such as those developed by the EAU Robotic Urology Section (ERUS) using validated performance metrics, represent a promising model to ensure consistent, high-quality education while maintaining patient safety during the learning curve [19, 20]. 

This study has several limitations. First, the observational design, although prospective, precludes definitive causal inference, and residual confounding cannot be excluded. Although the multivariable models were adjusted for hospital type, clustering at the individual center level was not accounted for, and residual center-specific effects related to surgical expertise, institutional workflows, or case mix cannot be fully excluded. Nevertheless, this risk was mitigated through prespecified multivariable adjustment using clinically relevant covariates. Second, the registry does not include granular information on individual surgical steps or the exact proportion of the procedure performed by the trainee. Because supervision by an attending surgeon is always present, the supervising surgeon may occasionally perform specific technically demanding maneuvers. By design a registry cannot capture the precise distribution of individual operative steps. However, teaching status was a mandatory, prospectively recorded variable reflecting the designated primary operator for each procedure, ensuring complete and standardized exposure classification across the cohort and supporting the validity of the comparison. Third, functional outcomes such as continence and potency, as well as early and late oncologic outcomes, were not analyzed because both were considered outside the predefined scope of this study, which specifically focused on perioperative outcomes and surgical safety. However, we acknowledge that these outcomes are highly relevant for patients and represent important measures of surgical quality. Nevertheless, all patients remain enrolled in the ongoing national registry, which prospectively collects follow-up data and will enable future longitudinal analyses specifically addressing functional recovery and long-term oncologic outcomes.

Finally, generalizability may be limited to the Swiss healthcare system, where training centers follow similar quality standards; this consistency, however, reduces inter-center variability and strengthens the internal validity of the findings. Despite these limitations, the study also benefits from several important strengths. The registry is mandatory for all Swiss training centers as of 2025, ensuring comprehensive case capture and a high patient volume over a short timeframe. Diagnostic and therapeutic approaches across participating institutions closely reflect contemporary clinical standards. In addition, routine audits contribute to high data quality. Together, these factors support the robustness and reliability of the study’s findings and reinforce its relevance for real-world surgical practice.

## Conclusion

Teaching during radical prostatectomy is safe with no clinically relevant increase in complications and no adverse impact on pathological outcomes. Despite slightly longer operative times, the overall perioperative and pathological results were equivalent between teaching and non-teaching cases. These findings confirm that supervised surgical training is both feasible and necessary to ensure the continued development of skilled urologic surgeons. Integrating structured teaching within high-quality, well-supervised programs allows for the safe transfer of surgical expertise while maintaining excellent standards of patient care.

## Supplementary Information

Below is the link to the electronic supplementary material.

Supplementary Material 1

## Data Availability

Requests for access to deidentified data may be considered upon reasonable request to the corresponding author and subject to approval by the Swiss Urology Registry governance and applicable data protection regulations.
