# Supplementary material for: Teaching in radical prostatectomy is safe: a multicenter prospective registry analysis
Source: J Robot Surg. 2026 Mar 30;20(1):382. doi: 10.1007/s11701-026-03350-4 (PMC13661691; doi:10.1007/s11701-026-03350-4)
Supplement: Supplementary file 1 [file 11701_2026_3350_MOESM1_ESM.docx]

**Supplementary Appendix**

**Teaching in Radical Prostatectomy Is Safe: A Multicenter Prospective Registry Analysis**

**Table 1. Collaborators of the Swiss Urology Registry**

| **Investigator** | **Center** |
| --- | --- |
| Dr. Karim Kellou | Hôpital de Nyon, Nyon, Switzerland |
| Dr. med. Thomas Sautter | Uroclinic, Wetzikon, Switzerland |
| Dr. med. Wolfgang Schäfer | Spitalzentrum Oberwallis, Visp, Switzerland |
| Anja Rieger | Limmattalspital, Schlieren, Switzerland |
| Dr. med. Simone Brunschweiler | Bellevue Urology, Zürich, Switzerland |
| Dr. Thomas Tawadros | Hôpital Riviera-Chablais, Rennaz, Switzerland |
| Dr. med. Thomas Sautter | Uroclinic, Pfäffikon, Switzerland |
| Dr. Aron Cohen | Urocare AG, Küsnacht, Switzerland |
| PD Dr. méd. Daniel Nguyen | Hôpital Neuchâtelois, Neuchâtel, Switzerland |
| Dr. med. Gautier Müllhaupt | Spital Thun (SpitalSTS AG), Thun, Switzerland |
| Dr. med. Stefan Preusser | Kantonsspital Schaffhausen, Schaffhausen, Switzerland |
| Prof. Ilaria Lucca | CHUV – Centre Hospitalier Universitaire Vaudois, Lausanne, Switzerland |
| Prof. Beat Roth | Inselspital, Bern, Switzerland |
| Dr. Thomas Luginbühl | Spital Uster, Uster, Switzerland |
| Dr. med. Roland Seiler | Spitalzentrum Biel, Biel, Switzerland |
| Astrid Bergundthal | Hirslanden Klinik Stephanshorn, St. Gallen, Switzerland |
| Soazig Olivin & Jérôme Chaptinel | Clinique de la Source, Lausanne, Switzerland |
| Dr. med. Katja Winzeler | Kantonsspital Baselland, Liestal, Switzerland |
| Prof. Massimo Valerio | Hôpitaux Universitaires de Genève (HUG), Genève, Switzerland |
| PD Dr. med. Räto Strebel | Kantonsspital Graubünden, Chur, Switzerland |
| PD Dr. Daniel Engeler | Kantonsspital St. Gallen (KSSG), St. Gallen, Switzerland |
| Prof. Daniel Eberli | Universitätsspital Zürich (USZ), Zürich, Switzerland |
| PD Dr. med. Tobias Zellweger | St. Claraspital, Basel, Switzerland |
| Prof. Dr. med. Agostino Mattei | Luzerner Kantonsspital (LUKS), Luzern, Switzerland |
| Prof. Dr. med. Hubert John | Kantonsspital Winterthur (KSW), Winterthur, Switzerland |
| Prof. Dr. med. Stephen Wyler | Kantonsspital Aarau, Aarau, Switzerland |
| Denise Bundi | Uroviva, Bülach, Switzerland |
| Julien Schwartz | Hirslanden Clinique Cecil, Lausanne, Switzerland |
| Dr. med. Stephan Bauer | Hirslanden Zentrum für Urologie (ZfU), Zürich, Switzerland |

**Table 2. Multivariable Logistic Regression for Any 30-Day Complication**

| **Variable** | **Adjusted OR** | **95% CI** | **p-value** |
| --- | --- | --- | --- |
| **Teaching operation** |  |  |  |
| No (Ref) | — | — | — |
| Yes | 1.41 | 1.09 – 1.82 | 0.008 |
| **D’Amico risk group** |  |  |  |
| Low (Ref) | — | — | — |
| Intermediate | 1.26 | 0.79 – 1.99 | 0.328 |
| High | 1.36 | 0.85 – 2.18 | 0.195 |
| **Age (years)** | 1.01 | 1.00 – 1.02 | 0.168 |
| **Body mass index (kg/m²)** | 1.02 | 1.00 – 1.04 | 0.032 |
| **ASA score** |  |  |  |
| ASA I (Ref) | — | — | — |
| ASA II | 0.98 | 0.70 – 1.38 | 0.915 |
| ASA III | 1.17 | 0.82 – 1.68 | 0.388 |
| **Prostate volume (mL)** | 1.00 | 1.00 – 1.00 | 0.887 |
| **Surgical technique** |  |  |  |
| Open/Converted (Ref) | — | — | — |
| Robotic | 0.44 | 0.34 – 0.55 | <0.001 |
| **Pelvic lymph-node dissection** |  |  |  |
| No PLND (Ref) | — | — | — |
| PLND performed | 1.47 | 1.17 – 1.84 | 0.001 |
| **Hospital type** |  |  |  |
| University (Ref) | — | — | — |
| Other | 0.49 | 0.38 – 0.63 | <0.001 |

**Table 3. Multivariable Logistic Regression for Severe Complications (Clavien–Dindo ≥ III)**

| **Variable** | **Adjusted OR** | **95% CI** | **p-value** |
| --- | --- | --- | --- |
| **Teaching operation** |  |  |  |
| No (Ref) | — | — | — |
| Yes | 0.62 | 0.24 – 1.36 | 0.283 |
| **D’Amico risk group** |  |  |  |
| Low (Ref) | — | — | — |
| Intermediate | 4.35 | 0.60 – 31.57 | 0.146 |
| High | 3.84 | 0.52 – 28.30 | 0.186 |
| **Age (years)** | 1.03 | 1.00 – 1.06 | 0.086 |
| **Body mass index (kg/m²)** | 1.02 | 0.99 – 1.05 | 0.197 |
| **ASA score** |  |  |  |
| ASA I (Ref) | — | — | — |
| ASA II | 1.27 | 0.45 – 3.56 | 0.651 |
| ASA III | 3.11 | 1.10 – 8.77 | 0.032 |
| **Prostate volume (mL)** | 1.00 | 1.00 – 1.01 | 0.398 |
| **Surgical technique** |  |  |  |
| Open/Converted (Ref) | — | — | — |
| Robotic | 0.44 | 0.26 – 0.73 | 0.001 |
| **Pelvic lymph-node dissection** |  |  |  |
| No PLND (Ref) | — |  | — |
| PLND performed | 1.45 | 0.83 – 2.52 | 0.193 |
| **Hospital type** |  |  |  |
| University (Ref) | — | — | — |
| Other | 1.40 | 0.57 – 3.41 | 0.464 |

**Table 4. Multivariable Logistic Regression for Need for Transfusion**

| **Variable** | **Adjusted OR** | **95% CI** | **p-value** |
| --- | --- | --- | --- |
| **Teaching operation** |  |  |  |
| No (Ref) | — | — | — |
| Yes | 1.56 | 0.36 – 4.60 | 0.478 |
| **D’Amico risk group** |  |  |  |
| Low (Ref) | — | — | — |
| Intermediate | 0.66 | 0.08 – 5.30 | 0.698 |
| High | 1.93 | 0.25 – 14.98 | 0.530 |
| **Age (years)** | 1.08 | 1.01 – 1.14 | 0.018 |
| **Body mass index (kg/m²)** | 1.02 | 0.97 – 1.09 | 0.414 |
| **ASA score** |  |  |  |
| ASA I (Ref) | — | — | — |
| ASA II | 0.30 | 0.09 – 0.95 | 0.041 |
| ASA III | 0.78 | 0.25 – 2.49 | 0.678 |
| **Prostate volume (mL)** | 1.00 | 0.98 – 1.01 | 0.776 |
| **Surgical technique** |  |  |  |
| Open/Converted (Ref) | — | — | — |
| Robotic | 0.19 | 0.09 – 0.41 | <0.001 |
| **Pelvic lymph-node dissection** |  |  |  |
| No PLND (Ref) | — | — | — |
| PLND performed | 1.38 | 0.40 – 4.78 | 0.609 |
| **Hospital type** |  |  |  |
| University (Ref) | — | — | — |
| Other | 2.33 | 0.29 – 18.72 | 0.425 |

**Table 5. Multivariable Logistic Regression for Positive Surgical Margins**

| **Variable** | **Adjusted OR** | **95% CI** | **p-value** |
| --- | --- | --- | --- |
| **Teaching operation** |  |  |  |
| No (Ref) | — | — | — |
| Yes | 0.99 | 0.81 – 1.22 | 0.960 |
| **D’Amico risk group** |  |  |  |
| Low (Ref) | — | — | — |
| Intermediate | 1.07 | 0.80 – 1.43 | 0.663 |
| High | 1.61 | 1.19 – 2.17 | 0.002 |
| **Age (years)** | 1.01 | 1.00 – 1.02 | 0.028 |
| **Body mass index (kg/m²)** | 1.03 | 1.01 – 1.05 | <0.001 |
| **ASA score** |  |  |  |
| ASA I (Ref) | — | — | — |
| ASA II | 1.03 | 0.81 – 1.31 | 0.798 |
| ASA III | 1.03 | 0.80 – 1.33 | 0.792 |
| **Prostate volume (mL)** | 0.99 | 0.99 – 1.00 | <0.001 |
| **Surgical technique** |  |  |  |
| Open/Converted (Ref) | — | — | — |
| Robotic | 0.83 | 0.68 – 1.01 | 0.068 |
| **Pelvic lymph-node dissection** |  |  |  |
| No PLND (Ref) | — | — | — |
| PLND performed | 1.09 | 0.94 – 1.27 | 0.248 |
| **Hospital type** |  |  |  |
| University (Ref) | — | — | — |
| Other | 1.29 | 1.03 – 1.61 | 0.025 |

**Table 6. Multivariable Logistic Regression for Positive Surgical Margins in the cT3–T4 Subgroup**

*D’Amico risk group was not included because it incorporates clinical T stage; instead, preoperative PSA and ISUP grade were modeled separately*

| **Variable** | **Adjusted OR** | **95% CI** | **p-value** |
| --- | --- | --- | --- |
| **Teaching operation** |  |  |  |
| No (Ref) | — | — | — |
| Yes | 1.23 | 0.69 – 2.18 | 0.478 |
| **Age (years)** | 1.01 | 0.99 – 1.03 | 0.472 |
| **Body mass index (kg/m²)** | 1.04 | 1.00 – 1.08 | 0.034 |
| **ASA score** |  |  |  |
| ASA I (Ref) | — | — | — |
| ASA II | 0.76 | 0.45 – 1.29 | 0.304 |
| ASA III | 0.81 | 0.45 – 1.45 | 0.482 |
| **Prostate volume (mL)** | 0.99 | 0.99 – 1.00 | 0.035 |
| **Surgical technique** |  |  |  |
| Open/Converted (Ref) | — | — | — |
| Robotic | 0.68 | 0.41 – 1.14 | 0.141 |
| **Pelvic lymph-node dissection** |  |  |  |
| No PLND (Ref) | — | — | — |
| PLND performed | 0.63 | 0.38 – 1.04 | 0.068 |
| **Hospital type** |  |  |  |
| University (Ref) | — | — | — |
| Other | 1.29 | 0.60 – 2.76 | 0.510 |
| **Preoperative PSA (ng/mL)** | 1.03 | 1.02 – 1.04 | <0.001 |
| **Preoperative ISUP grade** | 1.30 | 1.15 – 1.46 | <0.001 |

**Table 7. Multivariable Logistic Regression for Nerve-Sparing Performed**

| **Variable** | **Adjusted OR** | **95% CI** | **p-value** |
| --- | --- | --- | --- |
| **Teaching operation** |  |  |  |
| No (Ref) | — | — | — |
| Yes | 1.12 | 0.90 – 1.39 | 0.306 |
| **D’Amico risk group** |  |  |  |
| Low (Ref) | — | — | — |
| Intermediate | 0.67 | 0.47 – 0.95 | 0.026 |
| High | 0.33 | 0.23 – 0.47 | <0.001 |
| **Age (years)** | 0.94 | 0.93 – 0.95 | <0.001 |
| **Body mass index (kg/m²)** | 0.96 | 0.95 – 0.98 | <0.001 |
| **ASA score** |  |  |  |
| ASA I (Ref) | — | — | — |
| ASA II | 1.48 | 1.17 – 1.87 | 0.001 |
| ASA III | 1.07 | 0.83 – 1.37 | 0.608 |
| **Prostate volume (mL)** | 1.00 | 1.00 – 1.00 | 0.303 |
| **Surgical technique** |  |  |  |
| Open/Converted (Ref) | — | — | — |
| Robotic | 2.87 | 2.38 – 3.46 | <0.001 |
| **Pelvic lymph-node dissection** |  |  |  |
| No PLND (Ref) | — | — | — |
| PLND performed | 0.75 | 0.64 – 0.89 | <0.001 |
| **Hospital type** |  |  |  |
| University (Ref) | — | — | — |
| Other | 0.43 | 0.33 – 0.57 | <0.001 |

**Table 8. Multivariable Linear Regression for Operative Time**

| **Variable** | **Adjusted mean difference (min)** | **95% CI** | **p-value** |
| --- | --- | --- | --- |
| **Teaching operation** |  |  |  |
| No (Ref) | — | — | — |
| Yes | 31.2 | 25.6 – 36.8 | <0.001 |
| **D’Amico risk group** |  |  |  |
| Low (Ref) | — | — | — |
| Intermediate | 7.8 | 0.5 – 15.0 | 0.036 |
| High | 7.4 | −0.1 – 15.0 | 0.053 |
| Age (per year) | −0.02 | −0.25 – 0.21 | 0.889 |
| Body mass index (per kg/m²) | 1.25 | 0.86 – 1.64 | <0.001 |
| **ASA score** |  |  |  |
| ASA I (Ref) | — | — | — |
| ASA II | 13.8 | 7.7 – 19.9 | <0.001 |
| ASA III | 30.3 | 23.7 – 36.9 | <0.001 |
| Prostate volume (per mL) | 0.15 | 0.09 – 0.21 | <0.001 |
| **Surgical technique** |  |  |  |
| Open / Converted (Ref) | — | — | — |
| Robotic-assisted | 16.4 | 10.9 – 21.8 | <0.001 |
| **Pelvic lymph-node dissection** |  |  |  |
| No PLND (Ref) | — | — | — |
| PLND performed | 16.2 | 12.4 – 20.1 | <0.001 |
| **Hospital type** |  |  |  |
| University (Ref) | — | — | — |
| Other | −33.4 | −39.0 – −27.8 | <0.001 |

**Table 9. Multivariable Linear Regression for Lymph-Node Yield**

*Analysis restricted to patients undergoing pelvic lymph-node dissection*

| **Variable** | **Adjusted mean difference (nodes)** | **95% CI** | **p-value** |
| --- | --- | --- | --- |
| **Teaching operation** |  |  |  |
| No (Ref) | — | — | — |
| Yes | 0.55 | −0.34 – 1.43 | 0.226 |
| **D’Amico risk group** |  |  |  |
| Low (Ref) | — | — | — |
| Intermediate | 2.49 | 1.27 – 3.70 | <0.001 |
| High | 5.99 | 4.76 – 7.22 | <0.001 |
| Age (per year) | −0.04 | −0.08 – −0.01 | 0.022 |
| Body mass index (per kg/m²) | 0.15 | 0.09 – 0.21 | <0.001 |
| **ASA score** |  |  |  |
| ASA I (Ref) | — | — | — |
| ASA II | 0.62 | −0.26 – 1.49 | 0.170 |
| ASA III | 1.29 | 0.34 – 2.24 | 0.008 |
| Prostate volume (per mL) | 0.00 | −0.00 – 0.01 | 0.291 |
| **Surgical technique** |  |  |  |
| Open / Converted (Ref) | — | — | — |
| Robotic-assisted | −3.30 | −4.07 – −2.54 | <0.001 |
| **Hospital type** |  |  |  |
| University (Ref) | — | — | — |
| Other | −2.21 | −3.09 – −1.33 | <0.001 |
